# Does contingent biofeedback improve cardiac interoception? A preregistered replication of Meyerholz, Irzinger, Withöft, Gerlach, and Pohl (2019) using the heartbeat discrimination task in a randomised control trial

**DOI:** 10.1371/journal.pone.0248246

**Published:** 2021-03-16

**Authors:** Christian Rominger, Thilo Michael Graßmann, Bernhard Weber, Andreas R. Schwerdtfeger

**Affiliations:** 1 Institute of Psychology, University of Graz, Graz, Austria; 2 Otto Loewi Research Center, Section of Physiology, Medical University of Graz, Graz, Austria; Anglia Ruskin University, UNITED KINGDOM

## Abstract

Meyerholz, Irzinger, Withöft, Gerlach, and Pohl (2019) reported on a comparably large effect (*d* = 1.21) of a contingent biofeedback procedure on cardiac accuracy as assessed by the heartbeat tracking task. However, this task has recently been criticized as a measure of interoceptive accuracy. We aimed to replicate this finding by using the well-validated heartbeat discrimination task and to compare the biofeedback with a deep breathing and a control condition (viewing a film clip). The trial was preregistered at open science framework (https://osf.io/9fxn6). Overall, 93 participants were randomized to one of the three conditions and the heartbeat discrimination task was presented prior and after the 20-minutes training sessions. The study had a power of .86 to detect a medium-sized effect in the biofeedback group and a power of .96 to detect a medium-sized interaction of intervention group and time. A general tendency for improvement in heartbeat detection accuracy was found across intervention groups (*d* = 0.19, *p* = .08); however, groups did not differ significantly. In particular, there was no significant interaction of intervention group and time (*f* = .00, *p* = .98) and no reliable effect for the biofeedback group (*d* = 0.15, *p* = .42). One limitation is that a different, but well-validated task was used to quantify interoceptive accuracy. This study suggests that biofeedback might not improve interoceptive accuracy in the cardiac domain, but effects seem to depend on the specific task applied.

## 1. Introduction

In a previously published report, Meyerholz, Irzinger, Withöft, Gerlach, and Pohl [1] found that a brief 20-minutes contingent biofeedback procedure resulted in a large-scaled improvement of interoceptive accuracy as assessed with the heartbeat tracking task, which measures interoceptive accuracy by means of comparing the perceived heartbeats with actual heartbeats of a predefined time period (e.g., 25 sec, 45sec, etc. [2, 3]). Specifically, the biofeedback training was based on an animated heart symbol presented 200ms after R-wave detection and participants were instructed to press a button after a pre-defined number of heartbeats (2, 3 or 4 heartbeats). In the later training phases, the heart symbol was not presented. The authors conclude that cardiac biofeedback could improve interoceptive accuracy in the cardiac domain.

As the authors discuss themselves, the heartbeat tracking task has been criticized, because (implicit) knowledge about the own heartbeat could lead to better task performance [4, 5] and participants might achieve high accuracy without heartbeat perception, but accurate knowledge of heart rate [6]. Indeed, recent research seriously questions the validity of this task [7–9]. An alternative approach involves discrimination between true and false sensory feedback of individual heartbeats [10]. Although discrimination tasks have been criticized, because they may not solely warrant allocation of attention on internal and organismic cues [11], the integration of external and internal signals is a part of interoception [6] and fundamental for self-consciousness [12]. Heartbeat discrimination may be considered more valid [13, 14], since it is more robust against changes of (implicit) knowledge [4, 6, 10], and was suggested to be a prerequisite for heartbeat tracking [6]. Furthermore, some authors recommended the application of signal detection theory to study interoceptive accuracy [1, 7], which is implemented in discrimination tasks and allows to assess perceptual sensitivity separately from other non-perceptual factors [10]. Of note, interoceptive accuracy has been differentiated from self-evaluated assessments of subjective interoception and metacognition–that is the ability to discriminate correct from incorrect perceptual decisions [15, 16].

Therefore, the heartbeat discrimination task in combination with signal detection theory seems to be a good choice to replicate the training effects of Meyerholz et al. [1], since it allows to measure interoceptive accuracy independently from heartbeat-related knowledge [6] and the quantification of interoceptive metacognition—indicating the knowledge about own interoceptive performance [16]. The study-results may verify the validity of the reported contingent training effects and could indicate potential transfer-effects. Specifically, if the biofeedback training indeed enhances cardiac interoceptive accuracy, performance increases in the discrimination task could be expected. We used the very same training and passive control condition as Meyerholz et al. [1].

In order to extend the study of Meyerholz et al. [1], a further training condition was realized. Based on recent findings of an optimization of blood flow in brain areas associated with interoception (e.g., insula) due to deep nasal breathing [17], we aimed to examine the efficacy of a coherent breathing intervention on cardiac interoceptive accuracy. Of note, breathing at about 0.1Hz (i.e., 6 breaths per minute) has been associated with beneficial effects on physiology and psychological functioning [18, 19]. Slow breathing may induce resonance, meaning that metabolism in different physiological systems is synchronized and optimized [20, 21]. Furthermore, breathing seems to be important for corporal awareness [22], might change the focus of attention on internal body processes, and seems to activate an interoceptive network including the insula [17, 23], thus specifically coherent breathing potentially benefits interoception.

Taken together, firstly, we expected an increase in performance during the biofeedback training. Secondly, we hypothesized that the biofeedback training and the breathing condition would lead to an increase of interoceptive accuracy, in contrast to the control condition. Thirdly, we hypothesized that the training may increase the metacognition of participants.

## 2. Methods

### 2.1 Participants

**Fig 1:**
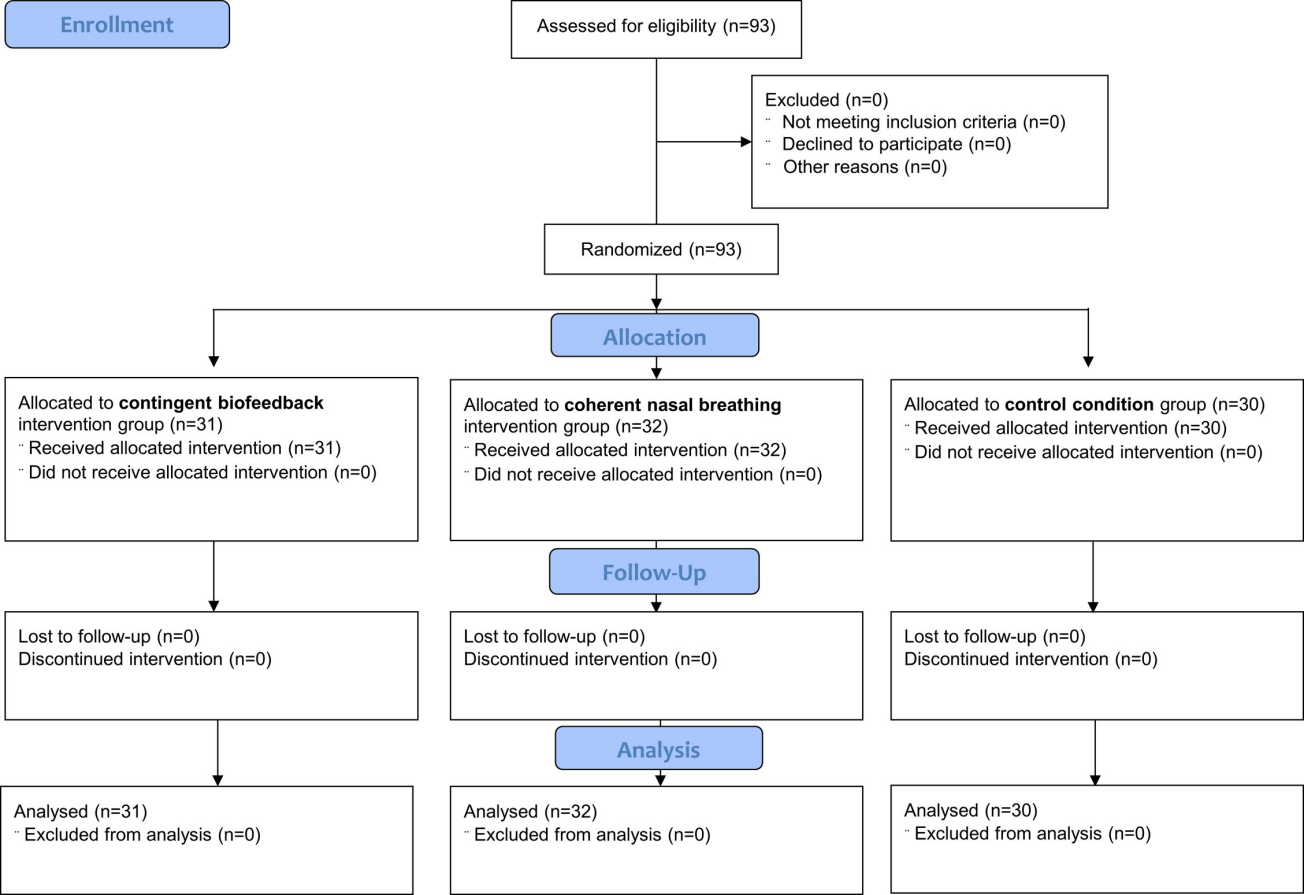
CONSORT flow chart illustrating the flow of participants through the study.

Based on the original study, which reported a large effect for the biofeedback group (*d* = 1.21) and a large effect for the between (group) x within (pre/post) interaction (*η*_*p*_^2^ = .24, *f* = 0.56), the required sample size for a mixed ANOVA 2 x 3 design given a power of .95 and moderate correlation between measures was N = 21 (G*power 3.192; [24]). However, due to overly large and biased effect size estimates of many (unregistered) primary studies [25, 26], we based sample size calculation on the assumption of small to medium effects (*d* = .30, *η*_*p*_^2^ = .05) with a power of .90. The resulting sample size was N = 90 (30 participants in each group). We strived to sample 93 individuals to account for dropouts. See Fig 1 for the flow of participants through the study. It should be noted that in the original study women (n = 74) were more prevalent than men (n = 26). Mean age was 23.95 years (*SD* = 4.18), which was slightly younger as compared to the original sample (*M* = 25.28, *SD* = 5.67). Due to technical problems the blood pressure of one participant and the heart rate of another participant were missing. Eligibility included a) being over 18 years and b) no regular yoga, meditation, mindfulness-related practice, or the use of heartbeat trackers. Ethics approval was granted by the Ethics Committee of the university of Graz (reference number: GZ.39/59/63 ex 2018/19), and the parallel randomised control trial was pre-registered in the Open Science Framework (https://osf.io/9fxn6). All participants gave their written informed consent.

### 2.2 Procedure and material

Participants were recruited at Graz from September to October 2019 via web, and flyer-based advertisements. They were randomly assigned to either of the three groups at a 1:1:1 ratio (see Fig 1). The second author generated the random allocation sequence by the Excel random number generation function, enrolled participants, and assigned them to the interventions, when they occurred at the laboratory. Although participants and the second author delivering the intervention could not be blinded to treatment assignment, the assessor conducting outcome assessments was blinded. Each condition lasted 20 minutes. Groups did not significantly differ on relevant variables (e.g., age, sex, education, pre/post heartrate, lifestyle variables). The only significant difference was found in the not-worrying subscale of the multidimensional assessment of interoceptive awareness (MAIA; [27]), where participants in the control condition showed slightly higher scores as compared to participants in the contingent biofeedback group (see Table 1).

**Table 1:** Descriptive data of the three experimental groups.

	Contingent biofeedback	Coherent breathing	Control condition	*F/Chi^2*	*p*
**Age**	24.13 (4.97)	23.75 (3.94)	23.97 (3.62)	0.064	.938
**Waist-to-hip ratio**	.78 (.06)	.81 (.06)	.79 (.16)	0.825	.441
**Trait anxiety (STADI)**	36.81 (7.44)	34.91 (9.31)	37.10 (8.56)	0.619	.541
**Factors of interoceptive awareness (MAIA subscales)**					
**Noticing**	4.13 (0.76)	3.98 (0.85)	4.23 (0.92)	0.639	.530
**Not-Distracting**	3.31 (0.89)	3.36 (0.89)	3.24 (0.77)	0.153	.858
**Not-Worrying**	3.26 (0.71)	3.50 (0.82)	3.82 (0.93)	3.613	.031
**Attention Regulation**	3.65 (0.64)	3.72 (0.94)	3.90 (0.75)	0.757	.472
**Emotional Awareness**	4.59 (0.65)	4.43 (1.02)	4.66 (0.83)	0.595	.554
**Self-Regulation**	3.27 (0.79)	3.66 (1.06)	3.55 (0.94)	1.437	.243
**Body Listening**	3.13 (0.78)	3.43 (1.03)	3.20 (1.05)	0.827	.440
**Trusting**	4.59 (1.00)	4.93 (0.99)	4.58 (1.06)	1.192	.308
**Pre-intervention HR (BPM)**	73.00 (10.33)	74.00 (11.04)	75.98 (12.74)	0.537	.586
**Post-intervention HR (BPM)**	69.47 (9.93)	71.58 (11.03)	72.20 (11.07)	0.506	.605
**BP systolic (mm/Hg) before/after baseline**	133.71 (18.06)/ 124.58 (15.41)	130.10 (11.88)/ 119.81 (10.44)	132.30 (17.87)/ 126.23 (17.74)	0.393/ 1.559	.676/ .216
**BP diastolic (mm/Hg) before/after baseline**	72.32 (8.43)/ 71.32 (7.91)	69.68 (7.20)/ 66.61 (7.24)	73.37 (10.04)/ 69.57 (10.77)	1.493/ 2.295	.230/ .107
**Gender**	10m/21w	17m/15w	18m/12w	5.14	.077
**Smoking**	26n/5y	26n/6y	22n/8y	1.13	.569
**Physical activity**	13n/18y	8n/24y	8n/22y	2.53	.283
**Education**	21h/10u	22h/10u	16h/14u	1.96	.376

*Note*. Smoking and physical activity were assessed by one question with a dichotomous yes (y) and no (n) answer. Education was dichotomous with high school (h) and university (u). BP = blood pressure. HR = heartrate. BPM = beats per minute. MAIA = Multidimensional Assessment of Interoceptive Awareness, STADI = State Trait Anxiety Depression Inventory.

#### 2.2.1 ECG

**Fig 2:**
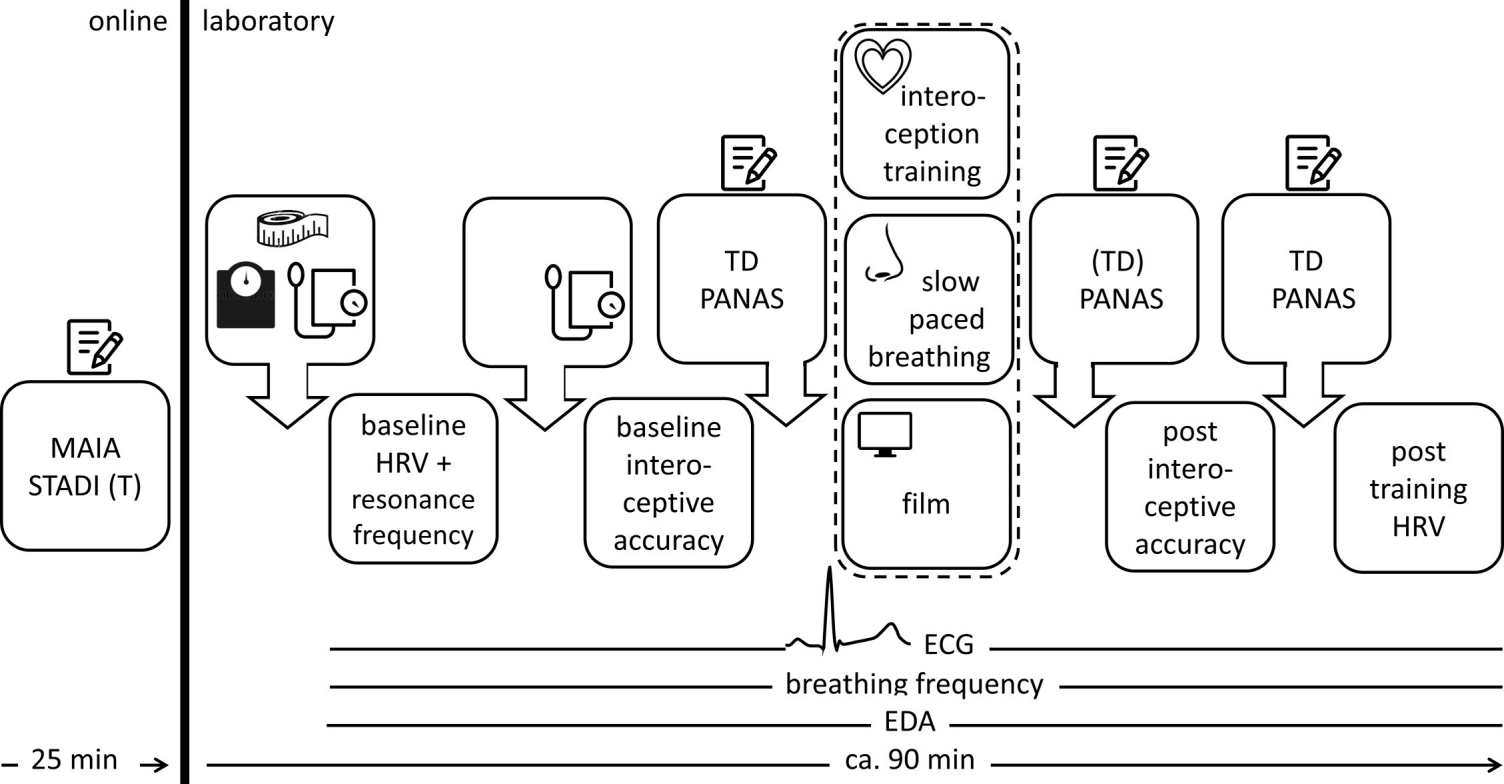
Procedure of the pre-registered replication study.

The ECG was recorded with a Biopac MP150 amplifier system (1000Hz) running AcqKnowledge 4.3 (standard lead II configuration). The R-waves were identified with the Accusync® 72 ECG Trigger Monitor, which sent R-wave contingent triggers to the computer running the biofeedback training and the heartbeat discrimination task (PsychoPy; [28]). Auditory stimuli were presented via stereo loudspeakers approximately 2m in front of the participants, who sat in a separated quiet and light attenuated room in a comfortable chair (1m in front of a computer screen). The instruction conformity was monitored by the experimenter via two cameras. A pre/post resting ECG with 3 minutes duration was recorded (Fig 2).

#### 2.2.2 Heartbeat discrimination task

Interoceptive accuracy was measured by the heartbeat discrimination task. Auditory playback of the participants’ heartbeats were presented with either a minimal (230ms) or prolonged (540ms) delay [10, 29, 30]. The task was to decide, after 10 tones (50ms duration; [29]), if the feedback accurately represented (was synchronous with) the own heartbeats or not. Thereafter the participants rated the confidence in perceiving their own heartbeat on a visual analogue scale form “total guess” to “complete confidence” [15]. First, one training block with 20 trials was conducted. The assessment of participants’ interoceptive accuracy consisted of 40 trials (in two blocks) for the pre/post intervention separately [31]. Interoceptive accuracy was indexed by d-prime [10, 29] with *d* = *z_hit rate_*−*z_false alarm rate_* (Z refers to the normal inverse cumulative distribution function).

Metacognition, that is knowing when making good or bad interoceptive decisions was determined by the area under a type II receiver operating characteristic curve [15, 16, 32]. We differentiated between positive and negative predictions and participants who thought to make good interoceptive decisions but did worse were indexed with a score lower than .5 (similar logic see [33]). With other words, participants who showed a negative association between subjective ratings and objective performance (systematically evaluating the interoceptive performance as good when showing poor accuracy) were indexed with scores lower than .5.

### 2.3 Experimental manipulation

#### 2.3.1 Contingent biofeedback training

The training was exactly conducted as described by Meyerholz et al. [1] and consisted of twelve initial trials to get familiar, followed by three blocks with 48 trials each. There was a short break of 15s in the middle of each block and a longer break between the blocks (max. 1min). In each trial, participants were asked to press a keyboard-button after 2, 3 or 4 consecutive heartbeats. Participant’s responses were classified as correct, if the button press fell within 200ms - 450ms after the final R-wave in the ECG. In the first 24 trials of each block, participants received a visual feedback (i.e., animated heart symbol) on their heartbeat (200ms delay) on the monitor. The intervals between the trials varied between one and four heartbeats. The number of heartbeats until button press and the intervals between the trials were pseudo-randomized. Participants received feedback after each trial in form of a checkmark (correct response) or a cross (false response) and after 24 trials by means of a percent-correct number.

#### 2.3.2 Coherent nasal breathing

Participants were instructed to breathe at their individual resonance frequency ([21], p. 23), which was determined during normal breathing at the baseline before the slow-paced nasal breathing intervention. A power spectral density analysis was applied to determine the individual resonance frequency. Specifically, the highest peak in the power spectrum within the HRV low frequency band (0.04–0.15Hz) was analyzed. If the highest peak fell above 0.12Hz or below 0.07Hz (19% of the subsample), respiratory frequency was set to 0.1Hz in order to comply with research favoring the benefits of slow breathing [18, 34]. The slow-paced nasal breathing consisted of seven blocks of two minutes each with one minute of rest in between (12 slow-paced training-breaths before intervention). Pacer stimuli were presented on a monitor: Inhalation was guided by an enlarging bar and exhalation by a lessening bar.

#### 2.3.3 Control condition

Similar to Meyerholz et al. [1], participants watched a nature documentary for 20 minutes.

### 2.4 Questionnaires

#### 2.4.1 Interoceptive awareness

The Multidimensional Assessment of Interoceptive Awareness (MAIA; [27]) is a self-report measures and was used to assess several factors of participants’ interoceptive awareness (German version, [35]). It is composed of 32 items, which are rated on a six-point Likert-Scale from 0 = *never* to 5 = *always*. The MAIA assesses eight concepts of interoceptive awareness (i.e., noticing, not-distracting, not-worrying, attention regulation, emotional awareness, self-regulation, body listening, trusting) with good psychometric properties (Cronbach’s α of subscales ranged from .66 to .87). In the present study, the subscales not-worrying (α = .44), not-distracting (α = .59), noticing (α = .61) showed low internal consistencies, while the other scales showed satisfactory Cronbach’s α of >.70.

#### 2.4.2 STADI

The State Trait Anxiety Depression Inventory (STADI; German version, [36]; based on [37]) was used to asses participants’ trait level of anxiety and depression by means of 20 items. All items were rated on a four-point Likert scale from 1 “nearly never” to 4 “nearly ever”. For the purpose of the present study we calculated a total score of the STADI (indexing negative affectivity) to control for differences between the intervention groups. The Cronbach’s α of this total score was .91.

### 2.5 Data analysis

The within-training effect was analyzed with an ANOVA for repeated measures and the factors block (block1/block2/block3) and phase (visual/no feedback). A mixed 3 (contingent biofeedback/coherent nasal breathing/control) x 2 (pre/post) ANOVA with the factors group and time was calculated to examine group specific effects on performance. Moreover, separate *t*-tests were performed in order to analyze the effect of interventions for each group separately. The alpha level was fixed at *p* < .05 (two-tailed).

## 3. Results

### 3.1 Manipulation check of biofeedback training

Comparison of the percent correct responses in the training blocks showed a significant main effects of block, (*F*(1.505,43.649) = 5.77, *p* = .011, *η*_*p*_^2^ = 0.17) and a main effect of phase (*F*(1,29) = 120.91, *p* < .001, *η*_*p*_^2^ = 0.81), but no interaction effect (*F*(2,58) = 0.925, *p* = .402, *η*_*p*_^2^ = 0.03). Similar to Meyerholz et al. [1], Bonferroni corrected pairwise comparisons indicated that the percentage of correct reactions improved significantly from block 1 (*M* = 55.37%, *SD* = 19.62) to block 3 (*M* = 65.90%, *SD* = 17.36; *p* = .027), which indicates the expected training effects. There were relatively more correct reactions in the phase with visual feedback (*M* = 76.45%, *SD* = 16.94) than without (*M* = 45.14%, *SD* = 14.93).

### 3.2 Effects of biofeedback training and slow breathing on cardiac interoception

Although the main effect of time was not significant (*F*(1,90) = 3.129, *Wilks Ʌ* = .966, *p* = .080, *η*_*p*_^2^ = .034), on a descriptive level performance increased from pre (*M* = 0.24, *SD* = 0.66) to post intervention (*M* = 0.39, *SD* = 0.71). Importantly, no significant interaction of group by time was found (*F*(2,90) = 0.020, *Wilks Ʌ* = 1.000, *p* = .980, *η*_*p*_^2^ = .000) as well as no main effect of group (*F*(2,90) = 1.717, *p* = .185, *η*_*p*_^2^ = .04). Separate t-tests for the two intervention conditions were non-significant (all *ps*>.293; see Table 2). The observed biofeedback training effect of *d* = 0.15 seems to be over 20 million times more consistent with the null hypothesis than with a large effect of *d* = 1.21, reported by Meyerholz et al. [1]. (The likelihood to observe a *d* = 0.15 when *d*_*true*_ = 0 is 0.277 and the likelihood to observe *d* = 0.15 when *d*_*true*_ = 1.21 is 1.372*10^−8^. The resulting likelihood ratio is therefore 20,189,504; see S1 Fig for the likelihood ratio distribution of observed data under an alternative vs. under the null hypothesis). In line with this, the Bayes factor of 0.26 strongly supports the null hypothesis for the observed biofeedback training effect [38].

**Table 2:** Observed means and standard deviations at pre-intervention and post-intervention for the intervention groups.

Measure	Group	Pre-intervention		Post-intervention		Within-group ESs (Cohen’s *d*, Pre to Post)		Between-group ESs (Cohen’s *d*, Intervention to Control)	
		Mean	*SD*	Mean	*SD*	*d*	(95% CI)	*d*	(95% CI)
D-Prime	Contingent biofeedback	0.37	0.77	0.49	0.76	0.15	-0.21–0.50	0.40	-0.12–0.90
	Coherent breathing	0.25	0.58	0.41	0.84	0.19	-0.16–0.54	0.25	-0.25–0.75
	Control condition	0.09	0.59	0.25	0.46	0.23	-0.14–0.59		
Metacognition	Contingent biofeedback	0.51	0.11	0.57	0.11	0.49	0.11–0.86	0.14	-0.37–0.64
	Coherent breathing	0.53	0.11	0.54	0.09	0.10	-0.25–0.44	-0.19	-.69–0.31
	Control condition	0.51	0.09	0.56	0.11	0.40	0.03–0.77		

*Note*. Between-group effect sizes were calculated for the difference between an intervention group (i.e., contingent biofeedback and coherent breathing) and the control condition group, separately. All effect sizes and CIs were estimated by means of the R package MOTE (version 1.0.2; [39]).

**Fig 3:**
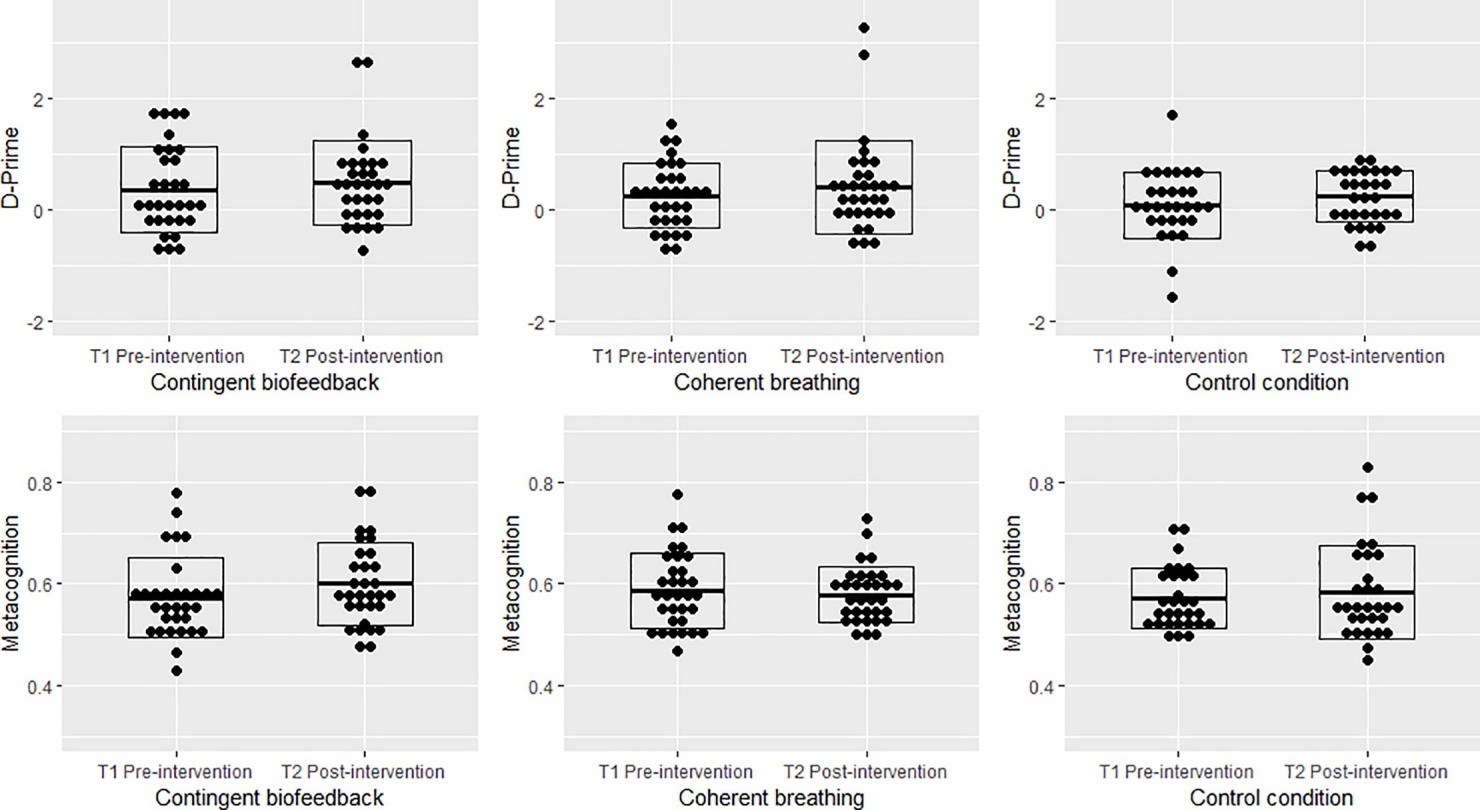
Interoceptive accuracy assessed with the heartbeat discrimination task from pre- to post-intervention (top row), separately for all the three groups (contingent biofeedback, coherent breathing, and control condition), which did not differ significantly from each other. Similar results are depicted for metacognitive performance (area under the type II receiver operating characteristic curve; bottom row).

Although the metacognitive performance revealed no interaction effect (*F*(2,90) = 1.348, *Wilks Ʌ* = .971, *p* = .265, *η*_*p*_^2^ = .03), it significantly increased from pre- to post-intervention (*F*(1,90) = 9.938, *Wilks Ʌ* = .901, *p* = .002, *η*_*p*_^2^ = .10; see Fig 3). The main effect group was not significant (*F*(2,90) = 0.128, *p* = .880, *η*_*p*_^2^ = .00).

Importantly, the change in interoceptive accuracy and metacognition from pre- to post-intervention was neither associated with performance in any training-block, nor with the training-effect from block1 to block3 (*ps*≥.216). None of the interoceptive awareness factors was associated with interoceptive accuracy or changes in thereof (*ps*≥.124). Only the subscale trusting showed a weak but significant association with metacognition before intervention (*r* = -.23, *p* = .029), but not after intervention (*r* = .07, *p* = .495).

## 4. Discussion

The aim of this research was to replicate the beneficial effect of a contingent biofeedback training on cardiac interoceptive skills [1]. Although we used the same biofeedback training and participants showed the expected increase of correct responses during this training, we did not observe the expected transfer-effect from the biofeedback training to the heartbeat discrimination task, which was expected to be an increase in interoceptive accuracy. This argues against the conclusion of Meyerholz et al. [1], thus suggesting that contingent cardiac biofeedback seems to not improve cardiac interoceptive accuracy (and neither metacognition).

Therefore, the findings of Meyerholz et al. [1] should be re-interpreted in terms of a within-task effect of training, which is not necessarily accompanied by an increase of cardiac interoception. As the authors discussed themselves, the direct feedback of participants’ heartbeats during the training might have changed participants’ knowledge leading to a better estimation of heart rate [40]. This interpretation is likely, since participants can achieve high scores in the tracking task without perceiving their heartbeats, but by guessing and estimating [4, 7, 8, 41]. In contrast, interoceptive accuracy derived from the heartbeat discrimination task has been considered largely independent of beliefs, knowledge, and the strategy to guess [4, 10]. The absence of an association pattern between the factors of the self-reported interoceptive awareness (MAIA) and the performance measure in the present study is in accordance with this [15, 42]. We only found one association between metacognition before intervention and the subscale trusting (out of 16; 8 subscales and two metacognition scores). Participants, who trusted their interoceptive perceptions more might be slightly more convinced to perceive (or not to perceive) their heartbeats, although their interoceptive accuracy is not different from others. This might indicate some validity of the measures. However, applying the type II receiver operating characteristic curve as a measure of metacognition has also been criticized [32]. Nevertheless, using the meta d-prime [32, 43] as an alternative index of metacognition resulted in a similar pattern of findings. All effects were non-significant (all *ps*≥.082).

Nevertheless, it should be emphasized that the present study does not falsify the findings of Meyerholz et al. [1], but rather complement them. Since this is a conceptual replication, the findings argue against the interpretation of an overall-effect on cardiac interoceptive accuracy [1]. If a contingent training would be effective over and above a mere training on the task, a transfer-effect should have occurred, and the training should have led to a significant increase in accuracy in the applied heartbeat discrimination task. The present null effect of the between-within interaction (*f* = .00) indicated no specific effect of biofeedback training. Furthermore, the observed intervention effect of *d* = .15 is much less consistent with the findings of Meyerholz et al. [1] as compared to the null-hypothesis. This convincingly indicates no general enhancing effects of the applied biofeedback training on cardiac sensation, which is in accordance with Phillips and colleagues [4]. These authors showed specific performance changes after (false) feedback-training in the heartbeat tracking task, while the discrimination task was unaffected. In a similar vein, Ring et al. [41] indicated that contingent and non-contingent feedback led to a comparable performance increase in the heartbeat tracking task. Until today, only Meyerholz et al. [1] reported a specific performance increase in a tracking task after a contingent training. However, the tracking task alone might not be a valid indicator for heartbeat perception, an assumption strengthened by the regular observation of a weak association between performance measures of the heartbeat tracking and the discrimination task [4, 15, 29, 40].

Similar to the feedback training, the coherent breathing condition showed no effects on interoceptive accuracy and metacognitive awareness. Probably, this training was too short or too difficult to change the blood-flow in brain areas related to interoception [17]. Nevertheless, the absence of a performance increase argues against a strong optimization effect of the current breathing intervention [18, 34] and future studies should probably apply trainings with longer durations [44] or should investigate coherent breathing as an acute strategy to modulate cardiac perception.

A further limitation of the present study is that beside the heartbeat tracking task also the heartbeat discrimination task has been criticized [45]. The major weakness of this task is that individuals largely differ in the way they perceive delayed feedback as synchronous with their heartbeats or not [45]. This might explain why only one of three (to four) participants can solve the heartbeat discrimination task adequately [45, 46]. Nevertheless, Wiens et al. [47] indicted that most individuals perceive intervals of about 200ms as synchronous and shorter as well as longer intervals more likely as asynchronous [48]. The applied discrimination task was grounded on this evidence.

### 4.1 Conclusions

Brief interventions such as biofeedback training and coherent breathing may not strongly alter cardiac interoceptive accuracy and metacognition. Potential training effects seem to depend on the specific task applied but not the phenomenon of cardiac sensations itself. Therefore, future studies should use different tasks assessing complementary aspects of cardiac interoception simultaneously in order to investigate interoceptive accuracy and metacognition in more detail.

### 4.2 Registration

This study was pre-registered in the Open Science Framework (OSF, https://osf.io/9fxn6;doi:10.17605/OSF.IO/9FXN6)

## Supporting information

S1 Dataset (XLSX)

S1 Fig Hypotheses chart. Distribution of likelihood ratios for observed data under an alternative hypothesis vs. under the null hypothesis. The graph is based on an adapted R script provided by Uri Simonsohn (see http://datacolada.org/appendix/78/hypchart%20post%202019%2009%2011.R). The inverse value of the presented likelihood ratio represents the ratio that data are more likely under the null hypothesis vs. under an alternative hypothesis (e.g., *d* = 1.21).
(DOCX)
