# Supplementary material for: Does contingent biofeedback improve cardiac interoception? A preregistered replication of Meyerholz, Irzinger, Withöft, Gerlach, and Pohl (2019) using the heartbeat discrimination task in a randomised control trial
Source: PLoS One. 2021 Mar 16;16(3):e0248246. doi: 10.1371/journal.pone.0248246 (PMC7963047; doi:10.1371/journal.pone.0248246)
Supplement: S1 Fig — Distribution of likelihood ratios for observed data under an alternative hypothesis vs. under the null hypothesis. The graph is based on an adapted R script provided by Uri Simonsohn (see http://datacolada.org/appendix/78/hypchart%20post%202019%2009%2011.R). The inverse value of the presented likelihood ratio represents the ratio that data are more likely under the null hypothesis vs. under an alternative hypothesis (e.g., d = 1.21). (DOCX) [file pone.0248246.s002.docx]

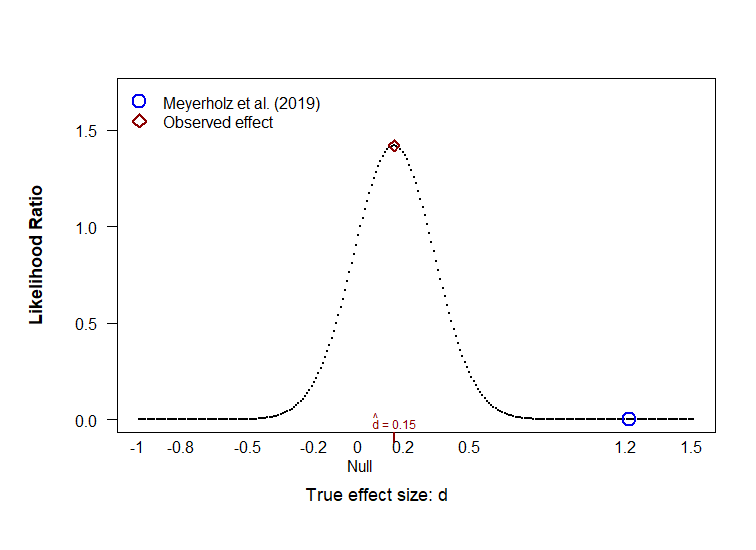


**S1 Fig. Hypotheses chart**. Distribution of likelihood ratios for observed data under an alternative hypothesis vs. under the null hypothesis. The graph is based on an adapted R script provided by Uri Simonsohn (see http://datacolada.org/appendix/78/hypchart%20post%202019%2009%2011.R). The inverse value of the presented likelihood ratio represents the ratio that data are more likely under the null hypothesis vs. under an alternative hypothesis (e.g., *d*=1.21).
